# Long-Term Durability and Variant-Specific Modulation of SARS-CoV-2 Humoral and Cellular Immunity over Two Years

**DOI:** 10.3390/ijms26168106

**Published:** 2025-08-21

**Authors:** Lilia Matei, Mihaela Chivu-Economescu, Laura Denisa Dragu, Camelia Grancea, Coralia Bleotu, Raluca Hrișcă, Corneliu Petru Popescu, Carmen C. Diaconu, Simona Maria Ruţă

**Affiliations:** 1 Stefan S. Nicolau Institute of Virology, 030304 Bucharest, Romania; lilia.matei@virology.ro (L.M.); denisa.dragu@virology.ro (L.D.D.); cgrancea@yahoo.co.uk (C.G.); coralia.bleotu@virology.ro (C.B.); carmen.diaconu@virology.ro (C.C.D.); simona.ruta@umfcd.ro (S.M.R.); 2 Romanian Academy Doctoral School, 010071 Bucharest, Romania; ralucamariahrisca@gmail.com; 3 Dr. Carol Davila’ Central Military Emergency University Hospital, 010825 Bucharest, Romania; 4 Faculty of Medicine, Carol Davila University of Medicine and Pharmacy, 050474 Bucharest, Romania; corneliu.popescu@umfcd.ro; 5 Victor Babes Hospital for Infectious and Tropical Diseases, 030303 Bucharest, Romania

**Keywords:** SARS-CoV-2, anti-SARS-CoV-2-specific antibodies, neutralization capacity, T-cell immunity, cytokine profile, long-term immunity, viral variants, longitudinal analysis

## Abstract

There is an increasing need to understand the long-term dynamics and quality of SARS-CoV-2 immune memory—both humoral and cellular—particularly with emerging variants. This study aimed to evaluate immune durability and variant-specific modulation through a longitudinal analysis of individuals with diverse SARS-CoV-2 exposure histories, over two years after infection and/or vaccination. The study involved assessing anti-spike IgG and IgA levels over time and analyzing their relationship with neutralizing activity against both ancestral and Omicron SARS-CoV-2 variants. Persistence of T cell responses was evaluated using intracellular cytokine staining (ICS) and activation-induced marker (AIM) assays. Anti-S IgG levels remained stable over time and increased after each immune stimulation, suggesting cumulative immune memory. Neutralizing capacity correlated strongly with IgG levels, showing long-term stability for pre-Omicron variants, but a moderate decline for Omicron. CD4^+^ and CD8^+^ T cell responses persisted across all groups, largely unaffected by Omicron mutations. However, cytokine profiles revealed subtle, variant-dependent changes. These findings underscore the durability of cellular immunity and the comparatively reduced robustness of Omicron-specific humoral responses. Such insights are crucial for understanding long-term protection against evolving SARS-CoV-2 variants and guiding public health strategies.

## 1. Introduction

SARS-CoV-2 vaccines have significantly reduced the severity of COVID-19 and its burden on the healthcare sector [1]. Nevertheless, due to the evolving nature of the virus, new immune-evasive variants—such as Omicron—have emerged, altering the long-term efficacy of both infection- and vaccine-induced immunity [2]. Therefore, understanding the durability and quality of immune responses following different types of exposure (vaccination, infection, or both) has become important to guide public health strategies.

It is well known that both humoral and cellular immune responses contribute to protection against SARS-CoV-2 [3,4]. Anti-spike (S) IgG antibodies, together with IgA and neutralization titers, are associated with protection from infection and reduced disease severity [5]. However, most studies have shown that antibody levels wane over time, and neutralizing capacity against Omicron subvariants is reduced due to the virus’s extensive mutations in the S protein [6]. Still, most studies on humoral immune responses cover a period of around one year [6,7], with some including follow-up at six months post-booster [8]. An exception is a study spanning three years [9]. However, none of these studies include analyses of cellular immune responses.

Several studies have addressed T cell responses and their role in immunological protection, as well as how the nature of acquired immunity affects the response [10]. However, most of these studies are generally conducted on small cohorts of participants with follow-up periods of up to 10 months [11,12], or offer a limited view, assessing only for IFN-γ levels measured by IGRA testing, even in larger cohorts [7].

However, most of these studies focus on only one type of immune response and the dynamics of immunity in the context of repeated exposure (either breakthrough infections or booster vaccinations), remain incompletely addressed, particularly when comparing responses to pre-Omicron and Omicron variants.

To overcome the current data limitations, we conducted a longitudinal study examining both types of immunity—humoral and cellular—in individuals with diverse virus exposure histories, more than two years after vaccination. This study builds upon our previous research, which investigated the longitudinal assessment of humoral immune responses in over 571 healthcare workers [13] and cellular immune responses in a subset of 20 participants [14], with follow-up periods of up to 6 and 12 months after BNT162b2 vaccination. By examining the dynamics of antibody and T cell responses over time (more than two years) and correlating this data with neutralizing capacity against both ancestral and Omicron variants over an extended period, we aim to characterize the durability and quality of SARS-CoV-2 immune memory. Our results provide insight into variant-specific influence on long-term immunity and may inform future strategies for variant-specific protection.

## 2. Results

### 2.1. Study Group Characterization

Participants were separated into three groups based on their immunity type at the time of enrollment: the infected group (unvaccinated individuals with natural acquired immunity following SARS-CoV-2 infection), the vaccinated group (individuals with vaccine-induced immunity), and the hybrid group (individuals with immunity resulting from both infection and vaccination). All vaccinated participants received two doses of the BNT162b2 mRNA vaccine, with an optional booster administered on average 8.7 months later (range, 4–14 months). All infected participants had mild or asymptomatic disease without complications. No participant reported chronic comorbidities or use of immunosuppressive therapy. The characteristics of the study cohort are presented in Table 1. Women were predominant in all groups. The mean age was comparable across the three groups, ranging from 43.9 (hybrid group) to 45.1 years (vaccinated group).

During the study, one participant had the second booster dose (BNT162b2 mRNA vaccine). Additionally, 18 infections with one of the circulating Omicron variants (BA.5 or XBB.1.5) were reported, with 50% of these cases being asymptomatic. The infections were detected based on an increase in anti-nucleocapsid (NCP) IgG antibody levels, which correlated with an increase in anti-S IgG antibody levels. Participants initially included in the vaccinated group, who were infected with SARS-CoV-2 virus during the study, were reclassified into the hybrid group after infection.

### 2.2. Dynamics of Anti-SARS-CoV-2 Antibody

**Figure 1:**
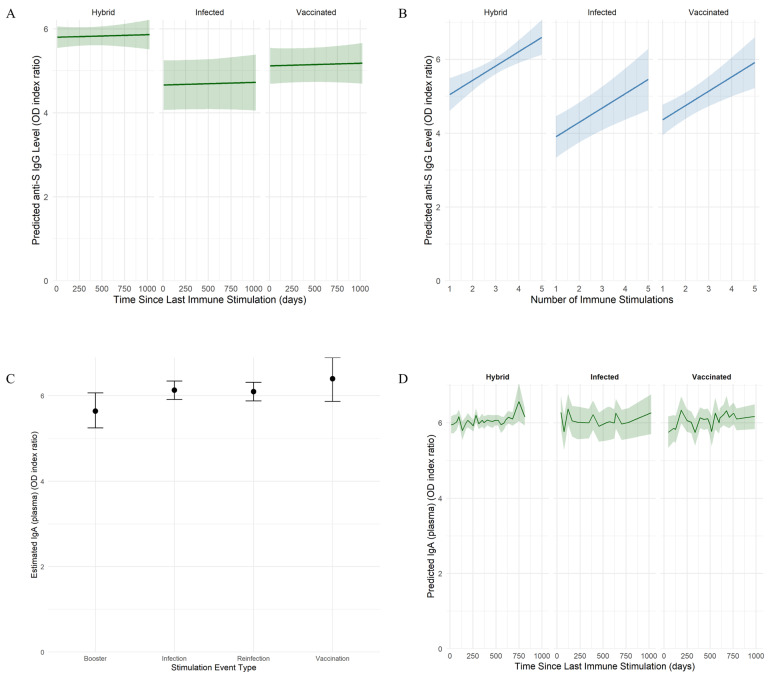
Model-based estimation of SARS-CoV-2-specific anti-S IgG and IgA dynamics. (**A**,**B**) Predicted anti-S IgG levels as a function of time since the last immune stimulation (**A**) and cumulative number of immune events (**B**). Individuals were grouped by immunity type into infected, vaccinated, and hybrid. Shaded areas represent 95% credible intervals based on a Bayesian model with skew-normal distribution. (**C**) Estimated plasma IgA levels by type of stimulation event. Immune stimulation events were grouped by type: vaccination, primary infection, reinfection, or booster immunization. Estimated marginal means (EEMs) of plasma IgA levels are shown with statistical credibility evaluated using 95% HPD intervals, based on an output of a Bayesian model with log-transformed Student’s *t*-distribution. (**D**) Predicted plasma IgA levels as a function of time since the last immune stimulation. Individuals were grouped by immunity type into infected, vaccinated, and hybrid. Shaded areas represent 95% credible intervals based on a Bayesian model with a log-transformed Student’s *t*-distribution.

To evaluate the persistence of anti-SARS-CoV-2-specific antibodies and their neutralization capacity, we fitted Bayesian mixed-effects regression models. The analysis of the anti-S IgG model revealed differences across immunity groups, being lower in infected (median: 3.50 optical density (OD) index ratio; 95% credible intervals (CI): 2.74–4.24) and vaccinated (median: 3.94 OD index ratio; 95% CI: 3.43–4.44) groups compared to the hybrid group (median: 4.62 OD index ratio; 95% CI: 3.81–5.43). Interestingly, anti-S IgG antibody levels remained largely stable over time since the last immune stimulation (vaccination or infection), following a similar pattern across all immunity groups (Figure 1A). In contrast, anti-S IgG levels increased with the number of immune system stimulations (an increase of 0.39 OD index ratio per event; 95% CI: 0.15–0.63), indicating a cumulative effect of prior exposures through vaccination or infection (Figure 1B). Additionally, substantial inter-individual variability was observed (standard deviation (SD): 0.83 OD index ratio; 95% CI: 0.64–1.04).

In the case of IgA model, the strongest predictor was the occurrence and type of recent immune stimulation (Figure 1C). Pairwise contrast confirmed higher plasma IgA levels observed after vaccination (median: 6.47 OD index ratio; 95% highest posterior density (HPD) interval: 5.92–7.00), infection (median: 6.21 OD index ratio; 95% HPD: 5.94–6.45) or reinfection (median: 6.17 OD index ratio; 95% HPD: 5.96–6.38) compared with booster (median: 5.72 OD index ratio; 95% HPD: 5.35–6.12). Still, the IgA plasma levels were similar. The effects of immunity type and elapsed time since the last immune stimulation were not associated with IgA variations, suggesting stable levels during the study timeframe (Figure 1D). Additionally, moderate inter-individual variability (SD: 0.30 OD index ratio; 95% CI: 0.18–0.43) was observed. Anti-NCP IgG levels declined rapidly over time, becoming undetectable in most cases by six months post-infection.

### 2.3. Neutralization Capacity Against Pre-Omicron and Omicron Variants

**Figure 2:**
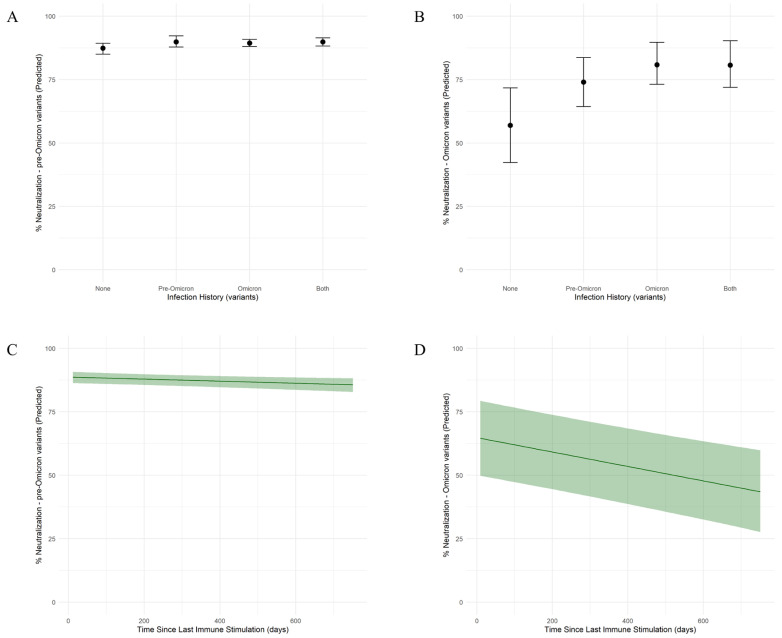
Model-based determinants of SARS-CoV-2 neutralization capacity against pre-Omicron and Omicron variants by infection history and time since the immune stimulation. (**A**,**B**) Predicted neutralization capacity (%) against pre-Omicron (**A**) and Omicron (**B**) variants by infection history. Individuals were grouped by their infection history into four categories: none (no infection), pre-Omicron infection only, Omicron infection only, or both (pre-Omicron and Omicron infections). Posterior medians are shown with 95% credible intervals based on a Bayesian model with skew-normal distribution. (**C**,**D**) Model-predicted neutralization capacity (%) against pre-Omicron (**C**) and Omicron (**D**) SARS-CoV-2 variants as a function of time since the last immune stimulation. Shaded areas represent 95% credible intervals based on a Bayesian model with skew-normal distribution.

The neutralization capacity was assessed against a pre-Omicron variant (Delta) and an Omicron variant. Among subjects without previous SARS-CoV-2 infection (vaccinated group), the neutralization capacity was higher against the pre-Omicron variant (median: 80.62%; 95% CI: 76.18–84.89) (Figure 2A) compared to the Omicron variant (median: 21.29%; 95% CI: –5.26–48.91) (Figure 2B). The neutralization of the pre-Omicron variant was positively associated with previous infections with both the pre-Omicron and Omicron variants (median: 83.10%; 95% CI: 80.67–85.81), as well as with pre-Omicron (median: 83.14%; 95% CI: 75.66–86.58) or Omicron variant only (median: 82.65%; 95% CI: 76.20–85.37). Still, the neutralization levels observed among all groups were similar. The neutralization of the Omicron variant was associated with previous infections with the Omicron variant, combined or not with infections with pre-Omicron variants (median: 45.15%; 95% CI: 27.37–61.71 for infections with the Omicron variant only and median 45.08%; 95% CI: 27.25–63.52 for infections with both variants) and at a lower level with previous infections with pre-Omicron only (median: 38.25%; 95% CI: 21.23–56.18).

The elapsed time since the last immune stimulation had a minimal negative effect for the pre-Omicron variant (Figure 2C), indicating limited waning over the studied time frame. In contrast, for the Omicron variant (Figure 2D), there is a gradual decline in neutralizing capacity over time, at a rate of 0.03% per day (95% CI: −0.04–−0.02).

**Figure 3:**
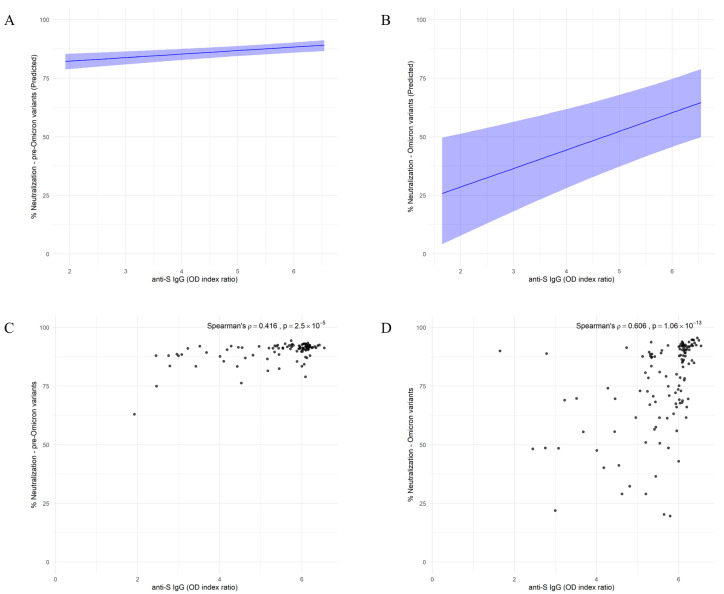
Model-based associations between anti-S IgG Levels and neutralization capacity against SARS-CoV-2 pre-Omicron and Omicron variants. (**A**,**B**) Model-predicted neutralization capacity against pre-Omicron (**A**) and Omicron (**B**) variants as a function of anti-S IgG levels. Shaded areas indicate 95% credible intervals based on a Bayesian model with skew-normal distribution. (**C**,**D**) Spearman correlations between anti-S IgG OD index ratio and neutralization capacity against pre-Omicron (**C**) and Omicron (**D**) variants. Spearman’s ρ and associated *p*-values are indicated on each panel.

The direct, unadjusted association between anti-S IgG levels and neutralization capacity was analyzed using Spearman’s rank correlation. A moderate positive correlation was observed between anti-S IgG levels and pre-Omicron variant neutralization (ρ = 0.416, *p* = 2.5 × 10^−5^) (Figure 3A). In contrast, a stronger correlation was detected between anti-S IgG levels and Omicron variant neutralization (ρ = 0.606, *p* = 1.06 × 10^−13^) (Figure 3B). The neutralization capacity of both used variants was strongly associated with anti-S IgG antibody levels, even after adjusting for a previous infection history and the time since the last immune stimulation. Neutralization capacity against the pre-Omicron variant increased by 1.49% for each 1 unit increase in IgG OD index ratio (95% CI: 0.78–2.23) (Figure 3C). In comparison, neutralization capacity against the Omicron variant increased by 7.82% for each 1 unit increase in IgG OD index ratio (95% CI: 4.06–11.53) (Figure 3D).

### 2.4. Persistence of SARS-CoV-2 Spike-Specific T Cells

**Figure 4:**
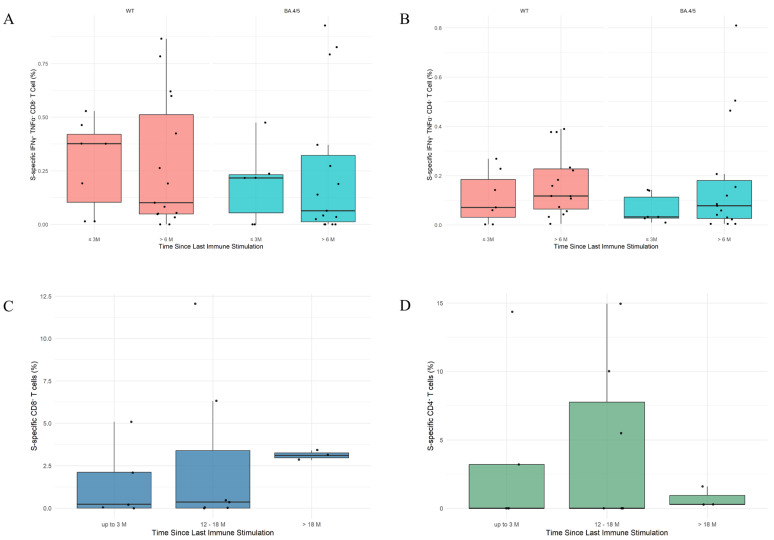
Dynamics of SARS-CoV-2 pike-Specific CD4^+^ and CD8^+^ T Cell Responses. (**A**,**B**) Frequency of CD8^+^ (**A**) and CD4^+^ (**B**) cytokine-secreting T cells by treatment (stimulation with either WT or BA.4/BA.5 SARS-CoV-2 spike peptides) and time. Individuals were grouped by elapsed time since the last immune stimulation (up to 3 months vs. more than 6 months). Each panel represents a different stimulation condition. (**C**,**D**) Frequency of CD8^+^ (**C**) and CD4^+^ (**D**) activated T cells over time following stimulation with BA.4/BA.5 SARS-CoV-2 spike peptides. Individuals were grouped by time since their last immune stimulation: up to 3 months, 12–18 months, and 19–24 months. Medians with interquartile ranges are presented.

The persistence of anti-S-specific T cells was assessed by evaluating intracellular cytokines and the activation-induced markers on T cells. To evaluate the dynamic cytokine production, the samples were stratified by immunity groups (hybrid, infected, and vaccinated) and categorized by elapsed time since the last immune stimulation (vaccination or infection) into two time groups: “up to 3 months” and “more than 6 months”. Comparisons of responses to in vitro stimulations with SARS-CoV-2 ancestral (WT) and BA.4/BA.5 variant-specific peptides were made within each immune and time group combination. The results revealed the presence of SARS-CoV-2-specific CD4^+^ and CD8^+^ T cells associated with TNF-α and IFN-γ secretion in all analyzed individuals, CD4^+^ and CD8^+^ T cells being reactivated by SARS-CoV-2 S-specific peptides from both WT and BA.4/BA.5 variants. The anti-SARS-CoV-2 cellular immune response was maintained for more than 6 months following the last immune system stimulation across all immune groups in both CD4^+^ and CD8^+^ T cells (Figure 4A,B), suggesting durable cellular immunity. Furthermore, when stratified according to infection history (no prior infection, infection with pre-Omicron variants, Omicron variants, or both), CD4^+^ and CD8^+^ T cell responses remained consistent, regardless of infection history for both WT and BA.4/BA.5 in vitro stimulations.

To evaluate the long-term persistence of T cell activation, we analyzed the activation markers expressed by CD4^+^ and CD8^+^ T cells induced by in vitro stimulation with SARS-CoV-2 BA.4/BA.5 S-specific peptides. The samples were stratified into three groups based on elapsed time since the last immune system stimulation: “up to 3 months”, “12–18 months”, and “19–24 months”. CD4^+^ and CD8^+^ T cells were activated in all samples, with no statistically significant differences between the time groups (Figure 4C,D).

### 2.5. Dynamics of Cytokine Memory Responses

To evaluate the persistence of memory T cell-associated cytokine responses, peripheral white blood cells (PBMCs) were stimulated with peptides specific for BA.1, a representative early Omicron subvariant, and BA.4/BA.5 and XBB.1.5, circulating subvariants during the study period, followed by quantification of cytokines. The relationships between Δ cytokine values (the difference between peptide-stimulated and unstimulated conditions) and the elapsed time since last immune stimulation were analyzed using Spearman’s rank correlation for each cytokine across each stimulation condition, and linear regression models were fitted. Summary statistics for Δ cytokine responses over time under specific-peptide stimulations including results of Spearman’s rank correlations (ρ, p, q) and linear-model slopes with 95% confidence intervals are detailed in Table 2.

**Figure 5:**
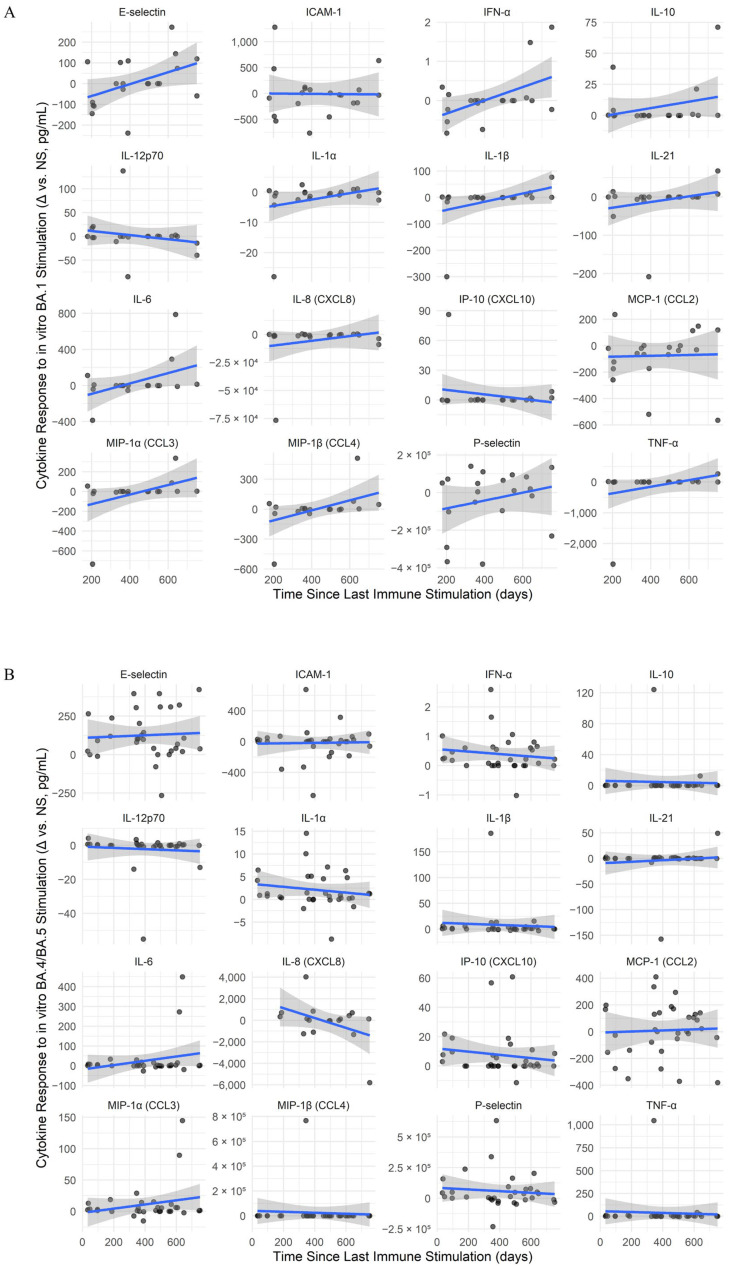

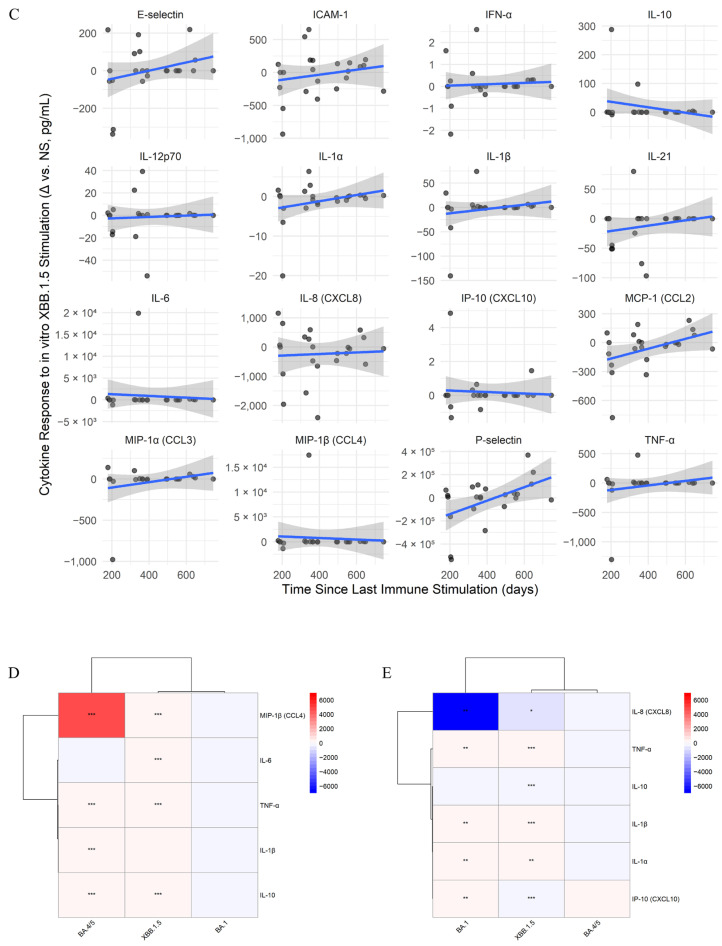
Longitudinal dynamics and SARS-CoV-2 variant-specific modulation of memory cytokine responses. (**A**–**C**) Cytokine responses over time for SARS-CoV-2 variant stimulations: BA.1 (**A**), BA.4/BA.5 (**B**), and XBB.1.5 (**C**). Line plots show antigen-specific cytokine responses (Δ = stimulated − unstimulated) over time since the last immune stimulation. Only paired samples from the same individual and time point were included to control for intra-individual variability. Solid lines represent linear model fits for each cytokine trend, and shaded areas indicate 95% confidence intervals. Points represent raw Δ values (pg/mL), calculated as stimulated minus unstimulated (NS) for the same individual at each time point. (**D**,**E**) Heatmaps of interaction effects between time since the last immune stimulation and prior infecting variant, BA.5 (**D**) and XBB.1.5 (**E**), on cytokine responses under different stimulation conditions. Each panel displays cytokines (rows) and stimulation conditions (columns), with color representing the interaction coefficient (red = increased response over time; blue = decreased response). Only cytokines with at least one statistically significant interaction (*p* < 0.05) are shown. Clustering reveals patterns of cytokine co-regulation and differential memory imprinting by variant. Significance levels: * *p* < 0.05, ** *p* < 0.01, *** *p* < 0.001.

Under BA.1 stimulation conditions (Figure 5A), TNF-α (Spearman’s ρ = 0.469) and E-selectin (Spearman’s ρ = 0.465) showed positive correlations with time. For BA.4/BA.5 stimulation (Figure 5B), IL-8 (CXCL8) showed a moderate negative correlation with time (Spearman’s ρ = −0.365). In the case of XBB.1.5 stimulation (Figure 5C), the highest correlation with time was for P-selectin (Spearman’s ρ = 0.358). Also, all stimulations induced a slight increase in IL-21 levels over time. However, despite these trends, all correlations were modest and non-significant after correction for multiple comparisons.

To assess how past infections influence cytokine secretion, linear models were fitted to analyze the combined effect between elapsed time since the last stimulation and infecting variant. In BA.5-infected individuals (Figure 5D), BA.4/BA.5 peptides triggered a marked increase in MIP-1β, while TNF-α, IL-10, and IL-1β increased slightly. XBB.1.5 peptides induced moderate MIP-1β and IL-6 increases. In XBB.1.5-infected individuals (Figure 5E), BA.1 peptides elevated TNF-α and slightly increased IL-1α, IL-1β, and IP-10, while IL-8 declined. XBB.1.5 peptides further raised TNF-α and modestly increased IL-1α, IL-1β, while IL-8 dropped more strongly. No significant effects were observed in individuals previously infected with Delta or BA.1. Overall, responses varied individually, with more notable increases in MIP-1β, especially following stimulation with peptides mimicking the infecting variant.

## 3. Discussion

Our longitudinal study provides an integrated analysis of humoral and cellular immune responses at 24 months following different SARS-CoV-2 immune exposures, highlighting the dynamics and variant-specific features of anti-SARS-CoV-2 immune memory.

The key findings of our study on the kinetics of the specific SARS-CoV-2 immune response are as follows: (a) anti-S IgG levels are stable over time and increased with each immune stimulation, indicating a cumulative effect of prior exposures; (b) the neutralization capacity was strongly associated with anti-S IgG antibody levels; (c) neutralization capacity against the pre-Omicron variant remained largely stable over time, showing minimal decline, whereas a mild but consistent decrease was observed for the Omicron variant; (d) CD4^+^ and CD8^+^ T cell responses persisted for at least 2 years after the last immune stimulation, regardless of infection history, indicating long-lasting cellular immunity to SARS-CoV-2.

The levels of anti-S IgG and IgA antibodies, along with their neutralizing titer, serve as markers for humoral immunity, being inversely correlated with the risk of symptomatic infections. High IgG levels can inhibit viral replication and promote viral clearance [15].

Our results indicated that anti-S IgG levels were influenced by the type of immunity (vaccinated, infected, or hybrid) and the number of previous immune stimulations. The highest level was observed in those with hybrid immunity type, followed by those from the vaccinated group and the infected group. These results are in accordance with ours [13,14] and others’ studies [7,8,9,12,16], which show synergistic effects of vaccination and infection on the humoral immune response.

Moreover, the observed positive effect of the number of immune system stimulations on anti-S IgG antibody levels supports the idea that repeated antigen exposures lead to a more persistent humoral response due to enhanced B-cell memory and antibody production [17,18]. Similar results were reported by Rubio et al. (2025), showing that repeated immunizations restore IgG antibody levels, often to higher levels [12]. Additionally, each new vaccine booster dose stimulates immune memory more effectively and reduces inter-individual variability, especially in cases with initial low IgG levels [19,20].

Our Bayesian mixed models indicated that IgG antibody levels remained relatively stable over time. According to previous studies, the anti-S IgG dynamic post-vaccination or post-infection is characterized by a more rapid initial decline in the first few months, followed by a more stable phase [9,21]. Repeated immunizations lead to a slower decline rate compared to that observed after previous immunizations, with IgG levels remaining relatively stable and showing only a minimal decrease over time [12]. In our study, the initial anti-S IgG waning effect reported by other studies was not observed, likely because the sampling time points did not capture this phase. Additionally, our modeling approach accounts for inter-individual variability and estimates relative changes from pre-existing levels, which may mask early declines. Moreover, our model integrates the cumulative effect of repeated immune system stimulations, through vaccination or breakthrough infections, reflecting the development of the anti-SARS-CoV-2 humoral immune response in individuals with diverse exposure backgrounds.

In light of previous conflicting reports on anti-NCP IgG persistence, our study demonstrated a rapid decline in anti-NCP IgG levels over time, with antibodies becoming undetectable in most cases by six months post-infection, consistent with our previous findings [13]. This contrasts with some studies reporting sustained anti-NCP IgG levels [22,23,24], while aligning with others that observed a significant decrease over time [8,25,26,27].

Regarding IgA levels, our findings indicated stability over time, with no significant influence from the type of immunity or the time elapsed since the last immune stimulation. IgA levels appear to be primarily affected by immune stimulation events, whereas vaccine booster doses exert a comparatively weaker effect. Anti-SARS-CoV-2 IgA antibodies are present in mucosal secretions and dominate the early neutralizing response against the virus. This early reaction is crucial for neutralizing the virus at entry points and preventing subsequent infection [28,29]. According to a study by Norton et al. (2023), high levels of IgA antibodies in plasma are associated with a reduced risk of infection, and their levels increase after booster doses or following breakthrough infections [30]. In our study, systemic IgA levels have been primarily affected by immune stimulation events, with vaccine booster doses exhibiting a weaker effect. This can be explained by our Bayesian mixed model, which estimates changes from pre-existing antibody levels, estimated marginal means (EEMs) representing relative variations rather than absolute ones. As such, in the case of individuals with boosters, the IgA basal level is already elevated due to previous vaccination and, in some cases, prior infection (in those with hybrid immunity).

On the other hand, the smaller post-booster IgA levels can also reflect saturation effects, as our model takes into consideration individual variability (via random intercept) and does not necessarily indicate poor responsiveness, but rather reflects the influence of pre-existing antibodies. Breakthrough infections have been associated with a more substantial effect on IgA levels, compared to vaccine booster doses [30], an effect that was also observed in our study group. According to Sheikh-Mohamed et al. (2022), individuals with breakthrough infections exhibit lower levels of systemic anti-SARS-CoV-2 IgA before infection compared to those who did not experience an infection [31]. Interestingly, in our study group, anti-S IgA levels remained stable over time and did not vary during the study period. This finding contradicts other studies, which report that IgA levels decrease rapidly during the first few months, after which they remain low, above the seropositivity cut-off, albeit with a slow decline [12].

We evaluated the neutralization capacity using a commercial SARS-CoV-2 surrogate virus neutralization test, rather than a live-virus neutralization test, which is considered the gold standard, having previously shown a strong correlation between the two methods [14]. Our results indicate that neutralization of pre-Omicron variants remains relatively strong, reflecting the durable immune response induced by ancestral strain-based vaccines or by previous infections. In contrast, neutralization of the Omicron variant was lower in the same groups, with a gradual decline over time. This aligns with current evidence suggesting Omicron exposure provides variant-specific immune boosting [32,33,34] and a faster waning after boosters [35,36], but also after infection with one of Omicron subvariants [33,36,37].

In our study, a moderate positive correlation was found between anti-S IgG levels and pre-Omicron variant neutralization, while a stronger correlation was observed for Omicron variant. The neutralization capacity against both variants remained strongly associated with anti-S IgG levels, even after adjusting for infection history and elapsed time since the last immune stimulation.

Overall, our results underscore the evolving landscape of antibody-mediated immunity in the context of emerging variants. Still, anti-SARS-CoV-2 humoral immune response in our cohort was influenced in a great measure by inter-individual variations, suggesting that host-specific factors, such as immune memory or genetic factors, may further modulate the immune response [38,39,40]. Other studies have reported that factors such as age, gender, Rh factor, and comorbidities such as diabetes, rheumatoid arthritis, HIV or oncological diseases are associated with anti-SARS-CoV-2 antibodies levels [13,22,41,42,43].

On the other hand, cellular immune responses play a crucial role in protecting against infections. The presence and activation of SARS-CoV-2-specific T cells, as part of the adaptive immune response, have been associated with milder forms of COVID-19 [44,45]. CD4^+^ T cells are essential for a protective humoral response and play a role in the maturation and proliferation of CD8^+^ T cells [46,47], while CD8^+^ T cells are involved in protection against severe forms of COVID-19 [48], contributing to the control of viral replication; their activation inversely correlates with the rate of viral decay [49].

The analysis of T cell responses in our study group revealed the persistence of anti-SARS-CoV-2-specific cellular immunity over time, concluding that the T cell response is less affected by Omicron mutations, as the immunodominant epitopes recognized by T cells are more evolutionarily conserved, mainly residing within regions that were minimally disrupted by mutations in emerging variants [50]. Responses to both pre-Omicron and Omicron peptides were similar across all samples, regardless of their immune history. Cytokine-secreting CD4^+^ and CD8^+^ T cells, as well as activation marker expression, showed no significant variability, indicating a consistent functional memory response.

However, previous infections seem to modulate the kinetics of cytokine responses. As such, BA.5 infections were associated with increasing MIP-1β responses, while XBB.1.5 infections showed rising TNF-α but declining IL-8 levels. Earlier variant infections led to more stable responses. Additionally, our results suggest a degree of cross-reactivity among T cells primed by previous infections, likely due to conserved epitopes across SARS-CoV-2 variants [51,52]. Breakthrough infections induce a rapid reactivation of memory T cell populations, both after previous infection and vaccination [49], and also diversify T cell memory repertoire [49,53]. In all individuals, regardless of stimulation condition, an increase in IL-21 is observed. IL-21 is a central mediator of adaptive immunity, enhancing both humoral and cellular responses by promoting B cell maturation and antibody production, as well as supporting effective and lasting CD8^+^ T cell responses [54,55].

The main limitation of our study is the small sample size, which may affect the statistical power of our results, especially after stratification by immunity type or infection history. To address this, we used robust statistical analyses, including Bayesian hierarchical models and random-effects analyses. Longitudinal samples were collected over a broad period, with variable intervals between immune events and sampling, determined by individual immune histories, which affected the evaluation of immune responses at specific time intervals. To mitigate this, we modelled time as a continuous variable, which allowed a flexible analysis of immune kinetics. Both ICS and AIM assays were used to capture complementary aspects of T cell memory in a subset of samples selected to ensure representation across immune groups and time points. Our analyses were focused only on identifying consistent trends across individuals. Nevertheless, these findings provide valuable insights into how the immune system responds to Omicron lineages.

Our study group did not include individuals with severe forms of COVID-19. Also, due to the widespread exposure in our region by 2022, fully unexposed controls were not available. However, our cohort is well characterized regarding infection history, comprising individuals who were monitored from the beginning of the SARS-CoV-2 pandemic. Even asymptomatic cases of infection were detected based on the individual dynamics of anti-NCP IgG levels, corroborated with other antibody dynamics (anti-S IgG and IgA). Consequently, our results primarily reflect immune responses associated with mild or asymptomatic infections and vaccination, and do not capture the immune dynamics related to severe forms of infection. Nonetheless, our cohort reflects the current situation of highly individualized vaccination schemes and infection history of the population from Romania, where only 42% of the total population has completed the primary COVID-19 vaccination series, and only 9% have received at least one booster dose [56].

Taken together, our findings underscore both the durability and complexity of SARS-CoV-2–specific cellular immunity. Immunity against pre-Omicron variants appears to be more stable over time. In contrast, humoral immunity following Omicron infection tends to be weaker, mainly due to extensive mutations in the spike (S) protein—particularly within the receptor-binding domain (RBD) and other critical regions. In contrast, cellular immunity has remained persistent over time, as T cell responses are less affected by Omicron mutations, given that the epitopes recognized by T cells are more evolutionarily conserved. However, specific cytokine pathways can show variant-specific modulation depending on prior infection history. Such differences might have implications for long-term immunity and the capacity to respond to emerging variants.

## 4. Materials and Methods

### 4.1. Study Group

To assess the durability of the immune response to SARS-CoV-2, we conducted a longitudinal study involving 68 individuals from September 2022 to December 2023. All subjects enrolled in the study signed a written informed consent form for participation, approved by the Ethics Committee of the Stefan S. Nicolau Institute of Virology (no. 1774/19 September 2022), and completed a brief questionnaire regarding current clinical symptoms and vaccination status at each visit. To reduce bias from self-reported data, which are susceptible to recall errors, misreporting, or incomplete responses, questionnaire information was cross-validated with medical records and laboratory findings whenever possible.

The study included subjects aged 18 years or older with a prior diagnosis of COVID-19 (positive RT-PCR test or presence of IgG anti-NCP antibodies) and/or vaccinated against SARS-CoV-2. Subjects with an incomplete vaccination schedule (e.g., infection after the first vaccine dose) or with fewer than two sample collections were excluded.

### 4.2. Sample Collection and Processing

Blood samples were collected in EDTA tubes from enrolled individuals every 3–4 months, from September 2022 to December 2023. Plasma was separated by centrifugation at 1500× *g* for 15 min and stored at −80 °C for further analysis. PBMCs were separated using Ficoll–Paque PLUS density gradient media (1.077 g/mL) (GE Healthcare, Buckinghamshire, UK), washed twice with phosphate buffered saline (PBS), and cryopreserved in AIM V medium (Gibco, Waltham, MA, USA) with 10% dimethyl sulfoxide (DMSO) at −80 °C.

### 4.3. ELISA Assays for SARS-CoV-2-Specific Antibodies

SARS-CoV-2 anti-S IgG and IgA and anti-NCP IgG antibody levels were determined using commercial ELISA kits Anti-SARS-CoV-2 ELISA (IgG), Anti-SARS-CoV-2 ELISA (IgA), and Anti-SARS-CoV-2 NCP ELISA (IgG) (EUROIMMUN Medizinische Labordiagnostika AG, Lübeck, Germany), according to the manufacturer’s recommendations. Briefly, plasma samples were diluted 1:100 and incubated in wells coated with recombinant SARS-CoV-2 S or NCP proteins. Specific IgG or IgA antibodies were detected by an immunoenzymatic reaction. Results were evaluated semiquantitatively by calculating a ratio between the optical density measured at 450 nm with reference at 630 nm (OD 450) of the sample and the OD 450 of a calibrator provided in the corresponding kit. The interpretation of the OD index ratio was as follows: <0.8—negative, ≥0.8 and <1.0—borderline, and ≥1.1—positive. The specificity and sensitivity of the used kits were 99.6% and 94.4% for the Anti-SARS-CoV-2 ELISA (IgG); 98.3% and >84.6% for the Anti-SARS-CoV-2 ELISA (IgA); and 99.8% and 94.6% for the Anti-SARS-CoV-2 NCP ELISA (IgG), respectively.

### 4.4. Surrogate Neutralization Capacity Assay

The neutralizing capacity of antibodies was assessed against two SARS-CoV-2 variants using the GenScript cPass™ SARS-CoV-2 Neutralization Antibody Detection Kit (Genscript, Piscataway, NJ, USA), a surrogate virus neutralization test (sVNT) that mimics the ability of antibodies to inhibit the interaction between the ACE-2 receptor and the Spike protein receptor binding domain (RBD), following the manufacturer’s recommendations. Plasma samples and positive and negative controls were diluted 1:10 and incubated at 37 °C for 30 min with enzyme-conjugated RBD proteins specific for either the Delta or the Omicron variants. The mixtures were transferred into ACE-2 coated wells and incubated for 15 min at 37 °C. After washing to remove HRP-RBD-antibody complexes, the enzyme substrate was added and incubated for 15 min at room temperature (25 °C). The neutralizing capacity of the antibodies was calculated using the formula:

% inhibition = [1 − (OD 450 sample/Mean OD 450 negative control)] × 100,

where OD 450 represents the optical density of the sample/control measured at 450 nm. An inhibition value greater than 30% was considered positive for neutralizing activity.

### 4.5. Intracellular Cytokine Staining (ICS) Assay

To assess the persistence of anti-S-specific T cells, longitudinal samples were selected from 20 individuals. The analyses were performed on mixed PBMC populations based on sample volume constraints, the need to preserve cell viability for multiple assays, and to capture in vivo-like cellular interactions.

The ICS assay was performed using the SARS-CoV-2 Prot S Complete T Cell Analysis Kit (Miltenyi Biotec, Bergisch Gladbach, Germany), designed for rapid detection of SARS-CoV-2–reactive T cells through intra- and extracellular staining of activation markers and cytokines. The kit includes antibodies against human CD3 (APC), CD4 (Vio Bright B515), CD8 (VioGreen), CD14 and CD20 (VioBlue), CD154 (APC-Vio770), TNF-α (PE), IFN-γ (PE-Vio770), along with a viability dye (Viobility 405/452 Fixable Dye).

PBMCs, previously stored at −80 °C, were thawed and incubated overnight in AIM V medium at 37 °C and 5% CO_2_. The following day, cells were centrifuged at 300× *g* for 5 min, resuspended in AIM V medium, and plated in 96-well plates at a density of 1 × 10^6^ cells/well. To assess SARS-CoV-2–specific responses, PBMCs were stimulated with either 2 µg/mL PepTivator SARS-CoV-2 Prot_S1 peptide pool (Miltenyi Biotec), 2 µg/mL recombinant SARS-CoV-2 S1 BA.4/BA.5 protein (R&D Systems, McKinley Place NE, MN, USA), sterile water with 10% DMSO (negative control), or 2 µL Cytostim (positive control). Cells were incubated at 37 °C and 5% CO_2_ for two hours, after which 2 µL Brefeldin A was added to inhibit cytokine secretion. Incubation continued for an additional four hours. After stimulation, cells were washed and stained with viability dye for 10 min at room temperature, then fixed with Inside Fix for 20 min and permeabilized with Inside Perm. Intracellular staining was performed using the antibodies provided in the kit, followed by washing and resuspension in MACS buffer (PBS with 0.5% bovine serum albumin and 2 mM EDTA). Samples were acquired using a MoFlo Astrios cell sorter (Beckman Coulter Life Sciences, Brea, CA, USA). Data were analyzed using Kaluza software version 1.3 (Beckman Coulter Life Sciences). The frequencies of specific cytokine-secreting CD4^+^ and CD8^+^ T cells were determined after subtracting the corresponding negative control and negative values were set to zero.

### 4.6. Activation-Induced Marker (AIM) Assay

To perform AIM assay a panel of antibodies was used, including anti-human CD3 (APC), CD4 (FITC) (clone OKT4, BioLegend, San Diego, CA, USA), CD8 (Brilliant Violet 510) (clone SK1, BioLegend), CD69 (APC-Vio770) (clone REA824, Miltenyi Biotec), CD137 (Brilliant Violet 650) (clone 4B4-1, BioLegend), and CD134 (OX40) (PE-Vio770) (clone ACT35, Miltenyi Biotec).

PBMCs stored at −80 °C were thawed and incubated overnight in AIM V medium at 37 °C and 5% CO_2_. The following day, the cells were centrifuged at 300× *g* for 5 min, resuspended in AIM V medium, and plated in 96-well plates at a density of 2 × 10^6^ cells per well. To assess the SARS-CoV-2–specific immune response, cells were treated with 2 µg/mL recombinant SARS-CoV-2 S1 protein BA.4/BA.5 (R&D Systems) and were incubated for 20 h at 37 °C and 5% CO_2_. Unstimulated cells were used as a negative control. The next day, the cells were resuspended in 100 µL MACS solution and centrifuged at 300× *g* for 5 min. Subsequently, cells were incubated in 100 µL Zombie UV Fix Viability kit for 30 min at 4 °C and washed with MACS solution. The cells were then stained with the antibody mixture by incubating for 30 min at 4 °C. Stained cells were washed and resuspended in MACS solution. Samples were acquired using a MoFlo Astrios cell sorter (Beckman Coulter Life Sciences). Data were analyzed using Kaluza software version 1.3 (Beckman Coulter Life Sciences). The frequencies of activated (CD137^+^ OX40^+^) CD4^+^ and (CD137^+^ CD69^+^) CD8^+^ T cells were determined after subtraction of the negative control and negative values were set to zero.

### 4.7. Cytokine Analysis

PBMCs stored at −80 °C were thawed and maintained overnight in AIM V medium at 37 °C and 5% CO_2_. The following day, the cells were centrifuged at 300× *g* for 5 min and then resuspended in AIM V medium. The cells were plated in 96-well plates at a density of 2 × 10^6^ cells per well and were treated with 2 µg/mL of recombinant S1 SARS-CoV-2 BA.1 protein (R&D Systems), recombinant S1 SARS-CoV-2 BA.4/BA.5 protein (R&D Systems), and recombinant S1 XBB.1.5 protein (R&D Systems). Unstimulated cells were used as a negative control. After 24 h of incubation, the supernatant was collected. Cytokine and chemokine levels were quantified using the xMAP technology with the ProcartaPlex Human Inflammation panel kit (ThermoFisher Scientific, Waltham, MA, USA) containing cytokines IFN-α, IFN-γ, IL-1α, IL-1β, IL-2, IL-4, IL-5, IL-6, IL-8, IL-9, IL-10, IL-12p70, IL-13, IL-17A (CTLA-8), IL-21, IL-22, TNF-α, and chemokines IP-10 (CXCL10), MCP-1 (CCL2), MIP-1α (CCL3), and MIP-1β (CCL4), following the manufacturer protocol. Briefly, 50 µL of the magnetic beads, coupled with antibodies specific to the targeted proteins, mixture was dispensed into each well of the plate and incubated with the supernatants at room temperature for 30 min with shaking, followed by overnight incubation at 4 °C, and an additional 30-min incubation at room temperature the next day. Unbound proteins were removed by washing, and quantitative detection of bound proteins was performed using streptavidin-PE–conjugated secondary antibodies. Data were acquired using a Bioplex 200 instrument (Bio-Rad, Hercules, CA, USA). Cytokine and chemokine concentrations were calculated by fitting sample values to the corresponding standard calibration curves using Bio-Plex Manager software (Bio-Rad, v6.1). Out-of-range low values were imputed as zero, and out-of-range high values were treated as missing. Antigen-specific responses (Δ cytokines values) were calculated as the difference between cytokine concentration in peptide-stimulated and unstimulated conditions.

### 4.8. Statistical Analysis

Categorical data were analyzed using Fisher’s exact test, and continuous variables were analyzed using Kruskal–Wallis tests with the Bonferroni correction for multiple comparisons. Pairwise comparisons were performed using Wilcoxon rank-sum tests with Benjamini–Hochberg correction for multiple testing. Where appropriate, continuous variables were represented by their median and interquartile range. A Spearman’s rank correlation test was used to analyze the correlation between variables. Statistical significance was set at *p* < 0.05.

Data distribution families were determined by fitting a series of Bayesian models using the same predictors and structure, and different likelihoods. The better models were selected using leave-one-out cross-validation (LOO), with the estimated higher expected log pointwise predictive density (elpd_loo) and the lower leave-one-out information criterion (LOOIC) used to indicate better predictive performance. All models passed the Pareto k diagnostic, suggesting reliable LOO estimates. Next, antibody levels and their neutralization capacity were analyzed longitudinally using Bayesian multilevel regression models with a skew-normal distribution for anti-S IgG antibodies and neutralization capacity against pre-Omicron and Omicron SARS-CoV-2 variants, and a log-transformed Student’s *t*-distribution for IgA antibodies. A random intercept was included for each subject to account for repeated measures within individuals. The convergence of each model was assessed using the Rhat statistic and effective sample sizes (ESS), with all Rhat values equal to 1.00 and high ESS, indicating good convergence and efficient sampling. Posterior estimates were summarized using 95% credible intervals (CI). The performance of each model was evaluated via LOO. To evaluate the effect of immune stimulation type on IgA levels, estimated marginal means (EMMs) and pairwise contrasts were computed. The credibility of observed differences was assessed using a 95% highest posterior density (HPD) interval. Regarding cytokine analysis, the combined effect between elapsed time since the last event and the previous infecting variant was analyzed by fitting linear models for each cytokine and stimulation condition, with interaction *p*-values adjusted for multiple testing using the Benjamini–Hochberg method.

All data were statistically analyzed in R (version 4.4.2) using the packages rstatix (version 0.7.2), brms (version 2.22.0), emmeans (version 1.10.5), and broom (version 1.0.7). Heatmaps were generated using the pheatmap package (version 1.0.12). All other plots were created using the ggplot2 package (version 3.5.1).

## Figures and Tables

**Table 1:** Study group characterization at enrolment.

Characteristic	Overall N = 68 ^1^	Hybrid N = 44 ^1^	Infection N = 8 ^1^	Vaccination N = 16 ^1^	*p*-Value ^2^	*q*-Value ^3^
**Sex**					0.4	>0.9
F	54 (79%)	34 (77%)	8 (100%)	12 (75%)		
M	14 (21%)	10 (23%)	0 (0%)	4 (25%)		
**Age**	44.2 ± 11.1	43.9 ± 11.3	44.1 ± 11.2	45.1 ± 11.0	0.9	>0.9
**Vaccination**	60 (88%)	44 (100%)	0 (0%)	16 (100%)	<0.001	<0.001
**Boosters**					0.004	0.019
0	31 (46%)	19 (43%)	8 (100%)	4 (25%)		
1	34 (50%)	22 (50%)	0 (0%)	12 (75%)		
2	3 (4.4%)	3 (6.8%)	0 (0%)	0 (0%)		
**Infections**					<0.001	<0.001
0	16 (24%)	0 (0%)	0 (0%)	16 (100%)		
1	36 (53%)	31 (70%)	5 (63%)	0 (0%)		
2	16 (24%)	13 (30%)	3 (38%)	0 (0%)		

^1^ N (%); Mean ± SD. ^2^ Fisher’s exact test; Kruskal–Wallis rank sum test. ^3^ Bonferroni correction for multiple testing.

**Table 2:** Summary statistics for Δ cytokine responses over time.

Cytokine	N ^1^ (Pairs)	ρ ^2^	*p*-Value ^3^ (Spearman)	*q*-Value ^4^ (Spearman)	Slope β ^5^ (pg/mL per Day)	95% CI (β)—Lower ^5^	95% CI (β)—Upper ^5^	*p*-Value (β) ^5^
**under BA.1 peptide stimulation**								
IL-12p70	20	0.465	0.039	0.208	0.286	0.010	0.562	0.043
IL-1α	20	0.523	0.018	0.208	−0.023	−0.072	0.027	0.351
IL-8 (CXCL8)	20	0.469	0.037	0.208	1.076	−0.429	2.581	0.150
IP-10 (CXCL10)	20	0.415	0.069	0.277	0.155	−0.015	0.326	0.071
IFN-α	20	0.310	0.183	0.367	0.002	0.000	0.003	0.022
IL-1β	18	0.328	0.183	0.367	0.574	−0.038	1.186	0.064
E-selectin	18	0.369	0.131	0.367	0.483	−0.063	1.029	0.079
ICAM-1	18	0.345	0.161	0.367	0.504	0.003	1.004	0.049
IL-10	19	0.290	0.228	0.405	0.074	−0.066	0.214	0.281
IL-21	20	0.264	0.261	0.417	0.031	−0.491	0.554	0.901
IL-6	20	−0.187	0.429	0.528	−0.044	−0.144	0.057	0.377
MCP-1 (CCL2)	20	0.192	0.418	0.528	0.011	−0.005	0.026	0.178
MIP-1α (CCL3)	20	0.208	0.380	0.528	209.962	−206.333	626.256	0.303
MIP-1β (CCL4)	20	0.068	0.777	0.828	0.026	−0.020	0.072	0.251
P-selectin	20	0.079	0.741	0.828	21.145	−22.725	65.016	0.325
TNF-α	20	0.047	0.845	0.845	−0.031	−1.200	1.138	0.956
**under BA.4/BA.5 peptide stimulation**								
IL-12p70	32	−0.339	0.058	0.748	−0.004	−0.022	0.015	0.695
IL-1α	32	−0.239	0.187	0.748	−0.003	−0.011	0.004	0.377
IL-8 (CXCL8)	17	−0.365	0.150	0.748	−4.586	−9.887	0.714	0.085
IP-10 (CXCL10)	32	−0.246	0.175	0.748	−0.011	−0.038	0.016	0.422
IFN-α	32	−0.175	0.339	0.929	0.000	−0.002	0.001	0.444
IL-1β	32	−0.171	0.348	0.929	−0.011	−0.070	0.049	0.712
E-selectin	32	0.007	0.971	0.971	0.043	−0.238	0.323	0.758
ICAM-1	32	−0.064	0.727	0.971	0.026	−0.362	0.414	0.892
IL-10	32	0.056	0.761	0.971	−0.004	−0.044	0.036	0.834
IL-21	32	0.014	0.938	0.971	0.015	−0.038	0.069	0.564
IL-6	31	−0.101	0.588	0.971	0.109	−0.056	0.274	0.186
MCP-1 (CCL2)	32	−0.022	0.904	0.971	0.040	−0.323	0.404	0.823
MIP-1α (CCL3)	31	0.032	0.865	0.971	0.033	−0.021	0.087	0.216
MIP-1β (CCL4)	32	0.134	0.466	0.971	−39.346	−286.152	207.461	0.747
P-selectin	32	−0.102	0.580	0.971	−68.545	−331.592	194.502	0.599
TNF-α	32	−0.022	0.903	0.971	−0.048	−0.383	0.288	0.775
**under XBB.1.5 peptide stimulation**								
E-selectin	23	0.236	0.278	0.736	0.217	−0.113	0.548	0.186
ICAM-1	23	0.181	0.407	0.736	0.381	−0.498	1.261	0.378
IL-21	23	0.250	0.251	0.736	0.045	−0.044	0.135	0.303
IL-6	22	0.204	0.362	0.736	−1.994	−13.650	9.662	0.725
IL-8 (CXCL8)	23	−0.179	0.414	0.736	0.266	−1.981	2.514	0.808
MCP-1 (CCL2)	23	0.230	0.292	0.736	0.510	0.006	1.015	0.048
MIP-1α (CCL3)	21	0.201	0.383	0.736	0.324	−0.266	0.914	0.264
MIP-1β (CCL4)	22	0.198	0.377	0.736	−1.537	−11.820	8.747	0.759
P-selectin	23	0.358	0.093	0.736	582.98	119.831	1046.135	0.016
IFN-α	23	0.080	0.716	0.747	0.000	−0.002	0.003	0.777
IL-10	23	0.114	0.603	0.747	−0.095	−0.255	0.064	0.228
IL-12p70	23	−0.073	0.739	0.747	0.006	−0.037	0.049	0.771
IL-1α	23	0.105	0.633	0.747	0.008	−0.004	0.020	0.188
IL-1β	23	0.158	0.470	0.747	0.044	−0.048	0.136	0.327
IP-10 (CXCL10)	23	0.071	0.747	0.747	0.000	−0.003	0.003	0.768
TNF-α	23	0.133	0.547	0.747	0.385	−0.374	1.143	0.304

Analyses used complete pairs. ^1^ The number of complete pairs per cytokine. ^2^ Spearman’s rank correlation. ^3^ Spearman’s two-sided *p*-values. ^4^ Benjamini–Hochberg correction for multiple testing. ^5^ Ordinary least-squares coefficient (Slope) from the model Δ ~ time with 95% confidence intervals (CI) and the corresponding (unadjusted) *p*-value.

## Data Availability

The original contributions presented in this study are included in the article. Further inquiries can be directed to the corresponding author.
